# Characteristics and causes of post-endoscopy Barrett’s neoplasia: Retrospective multicenter study

**DOI:** 10.1055/a-2781-6649

**Published:** 2026-01-22

**Authors:** Satoko Kako, Yugo Iwaya, Atsuhiro Hirayama, Takuma Okamura, Norikazu Arakura, Tomoaki Suga, Takayuki Watanabe, Akihiro Ito, Daichi Hara, Tadanobu Nagaya

**Affiliations:** 1 34808 Department of Medicine, Division of Gastroenterology and Hepatology, Shinshu University School of Medicine, Matsumoto, Japan; 2 73464 Department of Gastroenterology, Aizawa Hospital, Matsumoto, Japan; 3 26863 Department of Gastroenterology, Suwa Red Cross Hospital, Suwa, Japan; 4 73703 Department of Gastroenterology, Iiyama Red Cross Hospital, Iiyama, Japan; 5 631885 Department of Gastroenterology, Matsumoto City Hospital, Matsumoto, Japan; 6 13682 Department of Gastroenterology, Nagano Municipal Hospital, Nagano, Japan; 7 118108 Endoscopic Examination Center, Shinshu University Hospital, Matsumoto, Japan

**Keywords:** Endoscopy Upper GI Tract, Barrett's and adenocarcinoma, Quality and logistical aspects, Quality management, Image and data processing, documentatiton

## Abstract

**Background and study aims:**

Missed high-grade dysplasia (HGD) or adenocarcinoma in Barrett's esophagus (BE) may have serious consequences, although the attributes of post-endoscopy Barrett's neoplasia (PEBN) remain unexplored. We analyzed the characteristics of Barrett’s neoplasia (BN) eluding detection during screening endoscopy.

**Methods:**

We retrospectively reviewed endoscopic images of BN, including HGD and adenocarcinoma, diagnosed at six centers in Nagano prefecture. Eligible patients had index endoscopic images obtained 7 to 36 months before BN diagnosis. Causes of PEBN were classified as perceptual error, in which BN was missed despite images taken where it was eventually diagnosed, or exposure error, whereby no images were obtained in the area of BN development.

**Results:**

Among the 91 patients with BN, 31 were judged as having PEBN. The majority of PEBN cases were attributed to perceptual error (22 patients, 71%). Lesions within long-segment BE (LSBE) were significantly more likely to be overlooked due to exposure error (67% vs. 18%, *P* = 0.02), whereas lesions at the 0 to 3 o’clock position in short-segment BE (SSBE) tended to be missed due to perceptual error (76% vs. 33%, *P* = 0.04). Notably, 39% of perceptual error cases were misdiagnosed as esophagitis on index endoscopy. In the nine patients requiring surgery for PEBN, eight cases were attributed to perceptual error.

**Conclusions:**

PEBN occurring in LSBE was mostly overlooked because of inadequate observation, whereas PEBN at the 0 to 3 o'clock position in SSBE was frequently misdiagnosed as esophagitis. Bearing these results in mind may improve quality of endoscopic screening and reduce incidence of PEBN.

## Introduction

Barrett’s esophagus (BE) is a well-established risk factor for esophageal adenocarcinoma 1 . Although several gastroenterology-related societies have published guidelines to standardize screening and surveillance for Barrett's neoplasia (BN; including Barrett’s esophageal adenocarcinoma and high-grade dysplasia [HGD]) 2 3 4 , BN continues to be overlooked with concerning frequency. A retrospective analysis of more than 120,000 upper gastrointestinal endoscopies performed at four tertiary centers revealed a 6.4% miss rate for esophageal cancer along with an associated 2-year survival rate of only 20% 5 . Systematic reviews and meta-analyses have also indicated that up to 20% of BN cases are missed, with the proportion of post-endoscopy BN (PEBN) increasing from 5% in studies published before 2000 to 30% in studies appearing after 2015 6 . Accurately estimating the true prevalence of PEBN is important for devising optimal intervention strategies in tandem with current screening and surveillance approaches. Timely endoscopic detection of BN is also critical for preventing invasive BAC, thereby significantly improving patient outcomes 6 7 .

There is now a growing focus on gastrointestinal cancers detected after endoscopy, such as post-colonoscopy colorectal cancer (PCCRC), for which an incidence reduction has been associated with improved prognosis 8 . Similar findings have been published for PEBN, defined as BN detection following index endoscopy where no BN was initially identified 9 10 . Collecting and analyzing data on the course of endoscopic examinations, therefore, is pivotal for raising the quality of imaging procedures and diagnostic accuracy. However, specific endoscopic findings associated with PEBN and reasons for overlooking BAC remain unclear. This study sought to identify endoscopic characteristics and reasons for PEBN overlooked during screening endoscopy.

## Patients and methods

### Study design and patients

**Fig. 1:**
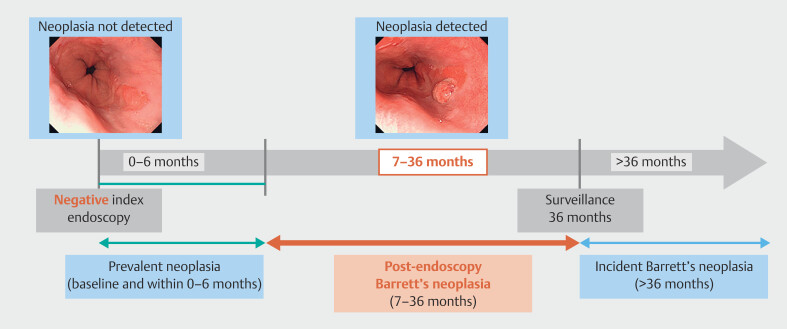
Definition of post-endoscopy Barrett’s neoplasia.

In this retrospective study, patients diagnosed and treated for HGD or superficial BAC at six secondary and tertiary referral centers in Nagano prefecture (population: 2 million) between January 2003 and December 2023 were eligible for inclusion. PCCRC has been defined as cancer detected after a negative colonoscopy in which no cancer was initially found 8 . Based on this definition, we established PEBN as HGD or BAC diagnosed within 7 to 36 months after index endoscopy last performed before BN diagnosis ( Fig. 1 ) in line with a previous report 9 . The 6-month interval from index endoscopy was to account for the possibility that if erosive esophagitis was present, a follow-up endoscopy or histological examination might have been necessary after the inflammation had healed. The upper limit was set at 3 years because American Society for Gastrointestinal Endoscopy (ASGE) guidelines have recommended that patients with non-dysplastic BE undergo surveillance endoscopy every 3 to 5 years 2 11 . Patients with available index endoscopy data who met these criteria were included for analysis.

This study was conducted following the Declaration of Helsinki and was approved by the Shinshu University Research Ethics Committee (registration no. 6227). Our institutional review board waived the requirement for informed consent, with patients provided the opportunity to opt out of the study on the hospital website.

### Endoscopy

Esophagogastroduodenoscopy (EGD) procedures were performed using video endoscopes (GIF-Q260/GIF-H260/GIF-H260Z/GIF-H290Z; Olympus Corp., Tokyo, Japan) and standard endoscopic video systems (EVIS LUCERA ELITE CV-290; Olympus Corp.). The decision to administer midazolam for sedation depended on patient preference. Premedication with scopolamine butyl bromide or glucagon was not given in any case. We diagnosed BE according to the Prague classification 12 , with long-segment BE (LSBE) defined as the longest diameter ≥ 3 cm and short-segment BE (SSBE) defined as the longest diameter < 3 cm. Patients who underwent endoscopy prior to establishment of the Prague criteria were classified by calculating C and M from the BE description as reported by the endoscopist. We endoscopically defined BE as ≥ 1 cm of columnar-appearing mucosa detected between the squamocolumnar junction and gastroesophageal junction, which was considered the oral end of the gastric folds or the distal end of the palisade vessels 13 14 . Biopsy collection was not routinely conducted according to the Seattle Protocol 2 15 ; rather, a targeted biopsy was performed upon lesion detection according to recent Japanese guidelines 16 17 . Although observation protocols and image documentation were not standardized across institutions, approximately four to 10 images of the esophagus were generally obtained during routine screening examinations at the participating centers. Additional images were captured when any area of concern was identified. Targeted biopsies were performed as standard practice when visible abnormalities were detected. Cancer morphology was judged according to the Paris endoscopic classification 18 . Main PEBN pathognomonic macroscopic type was classified according to the Paris classification as 0-I, 0-IIa, 0-IIb, or 0-IIc. Tumors with a circumferential involvement of less than two-thirds were evaluated based on the direction of the lesion's primary site. *Helicobacter pylori* status was determined by endoscopic findings according to the Kyoto classification 19 20 of gastritis, in combination with the results of *H. pylori* -related tests and interviews.

### Cause of error definitions

Endoscopic causes of misdiagnosis were classified into two categories.

**Fig. 2:**
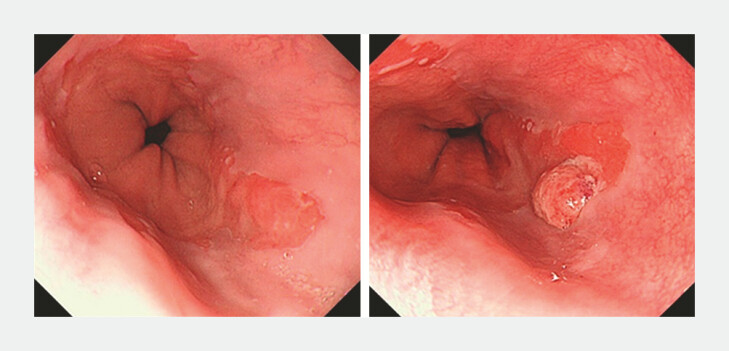
Representative images of perceptual error. a Index endoscopy showed a reddish and slightly elevated lesion at 5 o'clock in C0M1 SSBE. b Twelve months after index endoscopy, a tall ridge appeared. This case was classified as perceptual error. A biopsy specimen revealed adenocarcinoma.

**Fig. 3:**
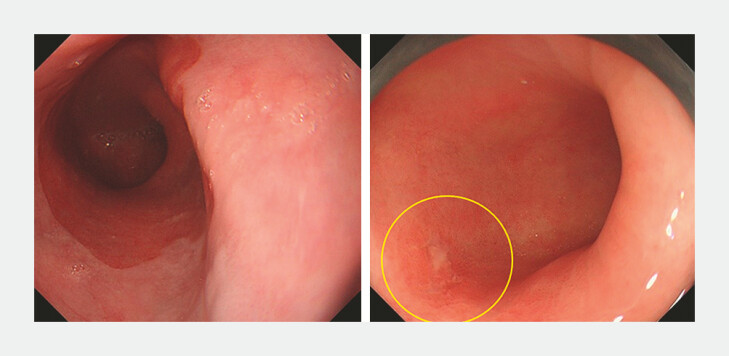
Representative images of exposure error. a During index endoscopy, only four images were taken and observation time was less than 1 minute, regardless of the C3M5 LSBE. b Twelve months later, EGD disclosed a 0-IIc lesion with an attached white coat at 6 o'clock in the LSBE (yellow circle). A biopsy specimen revealed adenocarcinoma.

Perceptual error was defined as a lesion that could be recognized retrospectively on index endoscopic images, but had not been diagnosed as BN ( Fig. 2 ). Exposure error was defined as no images taken of the area on index endoscopy in which the BN was eventually diagnosed ( Fig. 3 ).

Two board-certified endoscopists from the Japan Gastroenterological Endoscopy Society (YI and SK) independently reviewed the index endoscopy images to classify endoscopic causes of misdiagnosis. Any discrepancies in assessments were resolved by discussion between the observers. We quantified interobserver reliability using raw agreement and Cohen’s kappa (κ) with 95% confidence intervals (CIs).

### Pathological assessment

Pathological diagnoses were made by board-certified pathologists at each participating institution according to standard Japanese diagnostic criteria. Under the Japanese system, lesions diagnosed as “carcinoma” without submucosal invasion include both lesions equivalent to HGD and those equivalent to intramucosal adenocarcinoma in the Vienna classification. In contrast, the Vienna system classifies HGD (Category 4.1) separately from intramucosal carcinoma (Category 4.2), and only the latter is regarded as carcinoma 21 . To harmonize terminology for international readership, Japanese diagnoses were translated into the corresponding Vienna categories without altering the original pathological interpretation 22 .

### Statistical analysis

Categorical variables are presented as the count and percentage, whereas continuous variables are described as the mean or median. Differences between groups were analyzed using the Chi-squared test or Fisher’s exact test for categorical data. Student’s *t* -test or the Mann-Whitney U test were employed for comparing mean and median values, respectively, for continuous data. Statistical significance was determined as *P* < 0.05. All statistical analyses were performed using EZR (version 1.40; Saitama Medical Center, Jichi Medical University, Saitama, Japan), a graphic user interface for R (The R Foundation for Statistical Computing, Vienna, Austria).

## Results

### Baseline characteristics

**Fig. 4:**
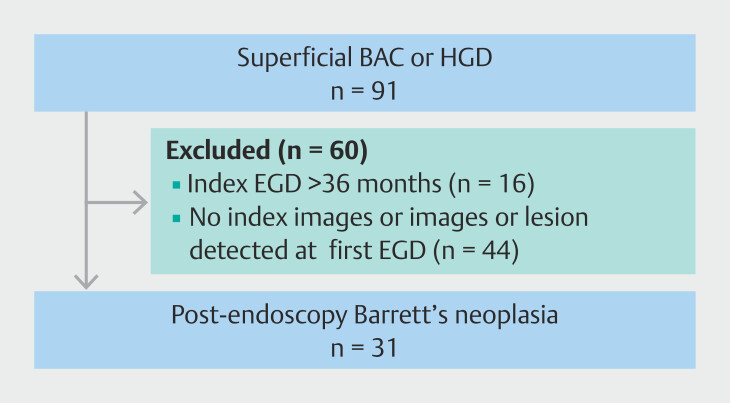
Flow diagram of patients who met study inclusion/exclusion criteria.

We identified 91 patients as having superficial BAC or HGD during the study period among the six participating institutions. Of the initial 91 patients, 16 were excluded because the index endoscopy had been performed more than 36 months earlier and 44 were excluded because either previous images were unavailable or the lesion had already been identified at the first endoscopy. As a result, 31 patients (31 lesions) met the strict definition of PEBN and were included in the final analysis ( Fig. 4 ). We explored narrower windows (6–24 and 6–18 months) post hoc, but these markedly reduced eligible cases and led to unstable estimates; therefore, we retained the 7- to 36-month window as primary. Clinical characteristics of those patients are summarized in Table 1 . Mean age at diagnosis was 64.7 years, with a male predominance (87%). The background of BE was 68% SSBE and 32% LSBE. Regarding tumor location, the majority of cases (61%) were found in the 0- to 3-o’clock position. Tumor depth analysis revealed 26% of patients to have T1b. Most PEBN cases were attributed to perceptual error (71%). For classification of perceptual vs exposure error, the raw agreement was 90% (28/31) and Cohen’s κ was 0.76 (95% CI, 0.50–1.00), indicating substantial agreement. Index endoscopy had been performed at 7 to 12, 13 to 24, and 25 to 36 months before BN diagnosis in 65%, 29%, and 6% of patients, respectively.

**Table:** **Table 1** Clinicopathological patient characteristics.

	N = 31
Mean age at diagnosis, years	64.7
Male	27 (87%)
*Helicobacter pylori* status	
Eradicated	3 (10%)
Infected	0 (0%)
Naïve	28 (90%)
Background Barrett’s esophagus	SSBE 21 (68%) LSBE 10 (32%)
Tumor location	0–3 o’clock 19 (61%) Other (4–11) 11 (35%) Circumferential 1 (4%)
Median tumor size, mm (range)	14.5 (3–107)
Treatment	
ESD	22 (71%)
Surgery	3 (10%)
ESD and additional surgery	6 (19%)
Macroscopic type: main type	0-I 2 (6%) 0-IIa 14 (45%) 0-IIb 4 (13%) 0-IIc 11 (36%)
Depth of tumor invasion	HGD/T1a 23 (74%) T1b 8 (26%)
Poorly differentiated component	7 (23%)
Vascular invasion Lymphatic invasion	1 (3%) 4 (13%)
Cause of misdiagnosis	Perceptual error 22 (71%) Exposure error 9 (29%)
Interval between diagnosis and index endoscopy	7–12 months 20 (65%) 13–24 months 9 (29%) 25–36 months 2 (6%)
ESD, endoscopic submucosal dissection; HGD, high-grade dysplasia; LSBE, long-segment Barrett’s esophagus; SSBE, short-segment Barrett’s esophagus.	

### Risk factors for perceptual error and exposure error

We next compared clinical characteristics of patients based on the cause of error ( Table 2 ). Univariate analysis showed that lesions at the 0- to 3-o’clock position were significantly more commonly missed due to perceptual error (73% vs. 33%, *P* = 0.04), whereas lesions in LSBE were significantly more likely to be overlooked due to exposure error (18% vs. 67%, *P* = 0.02). Notably, 39% of perceptual error cases were misdiagnosed as esophagitis on index endoscopy.

**Table:** **Table 2** Comparison of exposure error and perceptual error groups by univariate analysis.

	Perceptual error (N = 22)	Exposure error (N = 9)	*P* value
Mean age at diagnosis, years	64.8	68.1	0.84
Male	20 (91%)	7 (78%)	0.56
Background BE: LSBE	4 (18%)	6 (67%)	0.02
Tumor location: 0–3 o’clock	16 (73%)	3 (33%)	0.04
Median tumor size, mm (range)	15 (8–107)	12 (5–25)	0.20
Macroscopic type: main type (elevated:other)	10:12 (45%:55%)	6:3 (67%:33%)	0.50
Depth of tumor invasion (HGD+T1a:T1b)	14:8 (64%:36%)	9:0 (100%:0%)	0.08
Poorly differentiated component	5 (23%)	2 (22%)	0.66
BE, Barrett’s esophagus; HGD, high-grade dysplasia; LSBE, long-segment Barrett’s esophagus.			

### PEBN requiring surgery

Additional surgical resection was considered when non-curative factors were present, such as submucosal invasion, lymphovascular invasion, or poorly differentiated component according to the Japanese Esophageal Society Guidelines 17 . Nine patients with PEBN required surgery owing to a high risk of lymph node metastasis ( Table 3 ). All patients were male, with a mean age of 63 years. Eight lesions were attributed to perceptual error and one lesion was due to exposure error. Median lesion diameter was 20 mm and eight cases (89%) were located on the right wall apart from one circumferential lesion. Macroscopic type was 0-IIc in five cases and 0-IIa in four cases. Six lesions exhibited poorly differentiated components. All patients had undergone index endoscopy at 11 to 14 months before diagnosis of BN. Surgery was the initial choice for three patients, with additional surgery required after endoscopic resection in six patients. One patient who underwent surgery was found to harbor pathological stage IIIC. Multiple bone and lung metastases were detected 14 months after surgery and the patient succumbed to the primary disease 33 months postoperatively.

**Table:** **Table 3** Characteristics of cases requiring surgery.

No.	Treatment	Background Barrett’s esophagus	Tumor location	Type of error	Poorly differentiated component	Lymph node metastasis
1	ESD + surgery	SSBE	0–3 o’clock	Perceptual	+	-
2	ESD + surgery	LSBE	Circumferential	Perceptual	+	-
3	Surgery	SSBE	0–3 o’clock	Perceptual	+	-
4	ESD + surgery	LSBE	6 o’clock	Exposure	+	-
5	ESD + surgery	SSBE	0–3 o’clock	Perceptual	-	-
6	ESD + surgery	SSBE	0–3 o’clock	Perceptual	+	-
7	Surgery	SSBE	4–6 o’clock	Perceptual	-	-
8	ESD + surgery	SSBE	0–3 o’clock	Perceptual	-	-
9	Surgery	LSBE	0–3 o’clock	Perceptual	+	+
ESD, endoscopic submucosal dissection; LSBE, long-segment Barrett’s esophagus; SSBE, short-segment Barrett’s esophagus.						

## Discussion

To the best of our knowledge, this is the first report providing a detailed examination of clinical characteristics of patients and lesions in PEBN, as well as their associations with different causes of misdiagnosis. Although 65% of patients had undergone index endoscopy within a year in our cohort, 71% and 29% of lesions were missed due to perceptual and exposure errors, respectively. Univariate analysis showed that lesions at the 0- to 3-o'clock position were frequently missed due to perceptual error. Meanwhile, exposure errors were more likely to occur for lesions in LSBE.

In the clinical context, our results suggest that a careful differential diagnosis of inflammatory lesions is important in SSBE as the 0- to 3-o'clock position is a particularly common site for reflux esophagitis 23 24 . Progression from BE to BN initially involves inflammatory processes 25 26 . Therefore, endoscopic differentiation between inflammation and early neoplastic changes in BE can be challenging because both may present with similar visual features, including mucosal irregularities and nodularity. Presence of active inflammation can make histologic interpretation even more difficult by obscuring or imitating dysplastic changes. Accordingly, endoscopists should bear in mind that endoscopic diagnosis is complicated in cases with inflammation and consider reexamination and biopsy after the inflammation has subsided.

On the other hand, taking sufficient time to observe the entire area of LSBE appears essential because it is easy to miss lesions due to inadequate observation 27 . Risk of BN development in LSBE is high, and diagnosing tumors that manifest in LSBE is reportedly difficult 7 28 29 because BN distribution is highly focal and variable 30 31 . However, specific recommendations regarding the interval and method for surveillance in LSBE are not firmly established in Japan. European Societies recommend an inspection time of 1 minute per cm of circumferential extent of Barrett’s mucosa 4 , with a longer inspection time associated with enhanced BN detection 27 . More cases are needed to determine appropriate indicators for gauging endoscopist performance.

Among the nine surgical cases in our cohort, all but one were associated with perceptual error whereby the lesion was visible in endoscopic images but not recognized as neoplastic. Remarkably, all cases had undergone index endoscopy within 14 months before surgery, suggesting that these lesions were either subtle at the initial examination or had progressed rapidly during a short interval. This result highlights a critical diagnostic challenge in BN surveillance: subtle lesions with inconspicuous endoscopic features may harbor aggressive histological characteristics, such as the poorly differentiated components observed in 67% (6/9) of the cases. The combination of perceptual error and the aggressive nature of these lesions underscores the need for heightened awareness in BN surveillance. Endoscopists should remain vigilant to the fact that some cases of BN progress rapidly and exhibit high malignancy potential, making a proactive and meticulous approach essential for early detection and treatment.

There have been multiple reports about the usefulness of artificial intelligence (AI) for diagnosing neoplasia in BE 32 33 to improve sensitivity and specificity of non-specialists to those of experts with AI assistance 34 . Increased dissemination of AI is expected to help point out suspected lesions on-screen and decrease perceptual error. In order to reduce exposure error, AI systems that can improve quality indicators (QIs) are on the rise, such as those that can prompt identification of blind spots during EGD, provide grading scores for mucosal visualization, and measure inspection time. Although AI for lesion detection has progressed, there remain few systems to assess QIs, especially in the esophageal field 35 . Additional AI research is warranted to improve exposure error.

This study had several limitations. First, it was retrospective and contained a limited number of participants. Although alternative windows (6–24 and 6–18 months) were considered, the substantial loss of cases produced unstable models, limiting interpretability; future larger cohorts are warranted to reassess narrower intervals. Second, error type was determined through review of still images only. Reliable measurement of inspection times was not feasible in our retrospective multicenter setting, and thus, their correlation with exposure errors could not be assessed. Third, it was difficult to accurately distinguish between exposure error and newly developed cancer; however, because more than half of the patients in this study had undergone index endoscopy 7 to 12 months earlier, this issue was presumably minor. Fourth, our study did not include cases of random biopsy according to the Seattle Protocol 36 . This is because targeted biopsy based on endoscopic diagnosis is common in Japan 37 , with random biopsy not recommended by established guidelines 16 17 . When performing random biopsy, in addition to perceptual and exposure error, sampling error including intraobserver and interobserver variation in recognition of dysplasia by pathologists may occur 38 39 40 . Fifth, observation protocols and image documentation, including use of image-enhanced modalities, were not standardized across institutions. Lastly, although a multivariate analysis would have been desirable to identify independent predictors of perceptual and exposure errors, the limited number of PEBN cases (n = 31) precluded a stable model. Therefore, only univariate analyses were performed, and the present findings should be regarded as exploratory and hypothesis-generating. In contrast, the strengths of this study include patients from multiple institutions, from tertiary hospitals to primary healthcare facilities, which closely approximate real-world data. Furthermore, the detailed endoscopic and pathologic findings available for each case allowed us to precisely identify the types of diagnostic errors that are likely to take place.

## Conclusions

In summary, this study revealed that exposure error was more likely to occur in LSBE, whereas perceptual error was more prevalent in SSBE, especially in lesions located in the 0- to 3-o'clock direction. These results may provide valuable clues for endoscopists when diagnosing BE to enable earlier, less invasive treatments for BN.
